# Efficacy of Combined Vancomycin and Fosfomycin against Methicillin-Resistant *Staphylococcus aureus* in Biofilms *In Vivo*

**DOI:** 10.1371/journal.pone.0113133

**Published:** 2014-12-31

**Authors:** Jian Shi, Ning-Fang Mao, Li Wang, Han-Bo Zhang, Qian Chen, Hua Liu, Xun Tang, Tao Jin, Chong-Tao Zhu, Fu-Bing Li, Lin-Hui Sun, Xin-Ming Xu, Yong-Qing Xu

**Affiliations:** 1 Department of Orthopedics, Kunming General Hospital of Chengdu Military Region, Kunming, China; 2 Department of Orthopedics, Changhai Hospital, Shanghai, China; 3 Department of Pathology, Kunming General Hospital of Chengdu Military Region, Kunming, China; 4 Laboratory of Conservation and Utilization for Bio-resources and Key Laboratory for Microbial Resources of the Ministry of Education, Yunnan University, Kunming, China; 5 Department of Infection Control, Kunming General Hospital of Chengdu Military Region, Kunming, China; National Institutes of Health, United States of America

## Abstract

Infection by methicillin-resistant *Staphylococcus aureus* (MRSA) is a life-threatening condition, and formation of biofilms can lead to treatment failure in a clinical setting. The aim of this study was to demonstrate the *in vivo* bactericidal effects of a combination of vancomycin (VAN) and fosfomycin (FOS) against MRSA in a rat carboxymethyl cellulose-pouch biofilm model. The results of the time-kill assay showed that the combination therapy was capable of killing at low minimal inhibitory concentrations (MIC) (½× MIC VAN +1× MIC FOS and 1× MIC VAN + 1× MIC FOS). In the *in vivo* study, a synergistically bactericidal effect was observed when using the combination therapy on MRSA embedded in the mature biofilm model. In comparison with the untreated control group and the groups receiving either VAN or FOS alone, the rats treated with combination therapy had lower MRSA colony counts in exudates from the pouch, lower white blood cell and neutrophil counts, and C-reactive protein (CRP) in peripheral blood. Furthermore, histological analysis of the pouch wall indicated combination therapy resulted in disappearance of biofilm-like structures, marked decrease in necrosis, and formation of granular tissue. In conclusion, the combination of VAN with FOS had a synergistic bactericidal effect on chronic MRSA infection embedded in biofilm, providing an alternative approach to treating this condition.

## Introduction

Although antibiotics have played a major role in fighting infectious diseases, uncontrolled long-term or inappropriate use of antibiotics results in drug resistance and tolerant strains. Methicillin-resistant *Staphylococcus aureus* (MRSA) is undoubtedly one of the most prevalent and notorious examples of an antibiotic-resistant pathogen [1]. As a common, community-acquired and hospital-acquired Gram-positive pathogen, MRSA can cause a wide spectrum of diseases, including bacteremia, pneumonia, osteomyelitis and cellulitis, endocarditis, and septic shock and other infectious diseases [2], [3]. Facing the risk of severe MRSA epidemic, an increasing interest is aroused in uncovering the mechanisms of drug resistance and discovering new antibiotics and regimens to treat resistant strains.

Antimicrobial resistance is genetically based and mediated by the acquisition of extrachromosomal genetic elements containing resistance genes, which are transferred between bacteria via horizontal gene transfer [1]. A defining characteristic of MRSA is its ability to thrive in the presence of penicillin-like antibiotics, which normally inhibit the synthesis of the cell wall [1]. Resistance is mediated by the *mecA* gene, which stops β-lactam antibiotics from inactivating the enzymes (transpeptidases) critical for cell wall synthesis. Additionally, the development of resistance is associated with an acquired ability to form biofilms [4]. Biofilms are dense, highly hydrated clusters of bacterial cells that grow on living or inert surfaces and surround themselves with a polymer matrix composed of exopolysaccharides [5]. In the process of infection, *S. aureus* often attaches to host tissue or the surface of implants, and gradually forms a biofilm structure [6]. After biofilm formation, bacterial resistance to host immune responses and antibiotics can increase greatly. Bacteria in biofilms are up to 1000 times less sensitive than planktonic bacteria to antibiotics [7], [8].

For the past few decades, a number of newly developed antibiotics have been tested in laboratories and clinics. Among them, vancomycin (VAN) has served as the cornerstone of therapy against serious MRSA infections [7], [8]. The extensive use has elevated its minimal inhibitory concentration (MIC) and caused a MIC drift phenomena [9]. MRSA's biofilm formation can further increase its MIC markedly [10]. The polymerizable mucopolysaccharide secreted by MRSA at the biofilm surface limits penetration of antibiotics [11], increases the amount of time required for VAN to penetrate the biofilm, and decreases the permeability ratio. The long-term exposure of biofilms to drugs may promote adaptation to stress, thus further increasing bacterial resistance to antibiotics [12], [13]. Consequently, MRSA remaining in the biofilm are very difficult to eradicate, and the leftover bacteria can selectively thrive in the short term, thus leading to the failure of antibiotic treatment. Given that tolerant bacteria in the biofilm are genetically heterogeneous, combination therapy with two or more antibiotics has been the focus of multiple investigations [2], [10], [14]. However, combined therapy with VAN has rarely been reported [10], [15].

Fosfomycin (FOS), a broad spectrum antibiotic originally isolated from *Streptomyces fradicle* [16], penetrates tissues and has no cross resistance with other antibiotics. In addition, FOS has strong immunomodulatory activity [14]. Recently, several studies have confirmed that combinations of FOS and VAN have synergistic bactericidal effects on *Staphylococcus aureus in vitro* [17], [18], or in multiple MRSA strains embedded in biofilms [2], [19]. However, these results have not been confirmed *in vivo*. Therefore, the present study was designed to evaluate the combined effects of VAN and FOS *in vivo* on biofilm MRSA infection, using a carboxymethyl cellulose (CMC)-pouch biofilm model in rats. Results from the present study could provide a basis for future clinical studies aiming at demonstrating the efficacy of this combination therapy for MRSA infections.

## Materials and Methods

### 2.1 MRSA bacteria strain

MRSA strains KZ306, ATCC43300 and *S. epidermidis* strain ATCC35984 were used in this study. MRSA strain KZ306 was originally isolated from a patient suffering from chronic osteomyelitis, and was kindly provided by Clinical Laboratory Department, Kunming General Hospital of Chengdu Military Region. MRSA strain ATCC43300 and S. epidermidis strain ATCC35984 have been widely used in biofilm studies. All experiments followed the protocols are approved by Kunming General Hospital of Chengdu Military Region. Written consent was given. The Ethic Review Committee of Kunming General Hospital of Chengdu Military Region approved the animal use and experimental protocol. S. species were identified based on colony morphology, Gram's staining morphology and coagulase test results. MRSA was further confirmed based on the results of tube coagulase testing and bacterial growth on 6-µg/mL oxacillin salt-agar screening plates. MRSA was stored at −70°C before use, and recovered and cultured in soybean broth media, at 37°C for 20 h before use.

### 2.2 Antimicrobial susceptibility testing

The minimal inhibitory concentrations (MIC) of VAN (Eli Lilly, Seishin Laboratories, Japan) and FOS (Northeast Pharmacy, Shenyang, China) were determined for both strains according to “Performance standards for antimicrobial susceptibility testing, American's Clinical and Laboratory Standards Institute (CLSI); 16th informational supplement. Document M100-S16″, and titrated using a minute quantity broth dilution method [16]. For FOS testing, glucose-6-phosphate (25 mg/L; Sigma-Aldrich, St Louis, MO, USA) was added to the Mueller-Hinton medium.

### 2.3 Time-kill assay

The time-kill assay was carried out according to the CLSI methods [20]. In brief, the experiment included a control group, VAN group, FOS group, and a combination group (VAN and FOS). Newly prepared Mueller-Hinton broth medium (25 mL) without antibiotic was used as the control. Media (25 mL) with VAN or FOS alone, at the concentrations of ½×, 1×, 4× or 8× MIC, was added separately into corresponding tubes. Two antibiotics in combination at ½× MIC VAN + ½× MIC FOS, 1× MIC VAN + ½× MIC FOS, ½× MIC VAN +1× MIC FOS, or 1× MIC VAN + 1× MIC FOS were added to the corresponding tubes. The bacteria were diluted to 1×10^6^∼10^7^ CFU/mL, and 0.5 ml of the culture were collected at 0, 3, 6, 12, 24, 36 and 48 h after culture. Colonies were counted in 10-fold serially diluted 100-µL aliquots, plated on Muller-Hinton agar, and incubated at 37°C for 24 h. All experiments were performed in duplicate. The growth curve was plotted with average of bacteria counts at each time point.

The bactericidal activities were defined as ≥ 1000-fold reductions in colony-forming unit (cfu)/mL relative to the starting inoculum. Synergy was defined as a ≥ 100-fold decrease in cfu/mL for the combination compared with its most active constituent after 24 h of culture, with the number of surviving organisms in the presence of the combination being ≥ 100-fold cfu/mL below the starting inoculum [21].

### 2.4 Rat biofilm infection model

Forty-eight male Sprague-Dawley rats, weighing 250–300 g, were purchased from Laboratory Animal Center, Kunming General Hospital of Chengdu Military Region. The Ethic Review Committee of Kunming General Hospital of Chengdu Military Region approved the animal use and experimental protocol. The rats were acclimatized for 5–7 days prior to use in a room with a controlled temperature, relative humidity and a 12/12 h light-dark cycle. The rat sodium carboxymethylcellulose (CMC)-pouch MRSA biofilm infection model was established using the method described by Yasuda, et al [11], [22]. Air pouches were produced by the subcutaneous injection of sterile air (10 ml) into the intrascapular area of the back, with a 21-gauge needle, after the hair was trimmed off with a hair clipper. The next day (Day 1), 24 animals were randomly assigned into two groups for air pouch formation with two MRSA strains. Briefly, the CMC pouch was formed by injecting a mixture of 5 ml of 3% sodium carboxymethylcellulose (CMC) (Sigma-Aldrich, St Louis, MO, USA) and 5 ml of 1×10^7^ CFU/mL of MRSA strains KZ306, ATCC43300 or *S. epidermidis* strain ATCC35984 in saline. On Days 4 and 8, four rats that had been injected with one of the MRSA strains were euthanized, and the pouches were excised carefully, fixed with 10% formalin in 0.01 M PBS (pH 7.4), and embedded into paraffin wax blocks. Tissue sections of the pouches were stained with haematoxylin and eosin (HE) staining and with Gram staining method.

The remaining 16 rats for each MRSA strain were randomly divided into four groups (four rats per group): untreated control, VAN alone, FOS alone, and combined VAN and FOS.

### 2.5 Antibiotic therapy

The antibiotics were administered by daily intraperitoneal injection for 7 days, starting on Day 9 after the bacterial infection. The dosages were as follows: 100 mg/kg VAN, 200 mg/kg FOS, or a combination of both; the controls were injected with saline. At 24 h after the last drug injection, the rats were euthanized with a lethal injection of sodium pentobarbital (150 mg/kg); blood samples and exudates from the pouches were collected as described above.

### 2.6 Blood test and bacterial colony counts in pouch exudates

One day before and on Days 8 and 16 after the bacterial injection, the rats were anesthetized and blood samples (1.5–2.0 ml) were collected using sodium citrate anticoagulan from the retrobulbar plexus. White blood cell (WBC) counts and differentials were determined using an automatic blood cell analyzer (Minidray BC-5100, Minidray Co., China). C-reactive protein (CRP) was measured using an automatic serum analyzer (ViITROS Fusion 5.1, Johnson & Johnson, Rochester, NY, USA).

Samples of pouch exudates (100 µl each) were taken using a syringe on Days 4, 8 and 16 after the bacterial injection. The numbers of viable bacteria were counted, according to the methods reported by Morikawa, et al [22].

### 2.7 Histology

On Day 16 after the bacterial injection, pouch samples were obtained as described above, and all tissue samples were analyzed microscopically. Histological analysis of the pouch walls was carried out using HE staining and Gram staining for both strains.

### 2.8 Statistical analysis

The statistical analyses were accomplished using SPSS software (SPSS, Chicago, IL, USA). The data were presented as mean ± standard deviation. The differences among the experimental groups were compared by analysis of variance (ANOVA) followed by post-hoc Bonferroni test or ANOVA for repeated measurements. Furthermore, the Kruskal-Wallis *H*-test was used for analysis of the data with heterogeneity of variance. A *P*<0.05 value was considered statistically significant.

## Results

### 3.1 Bactericidal effects of VAN and FOS or combination of both *in vitro*

**Figure 1:**
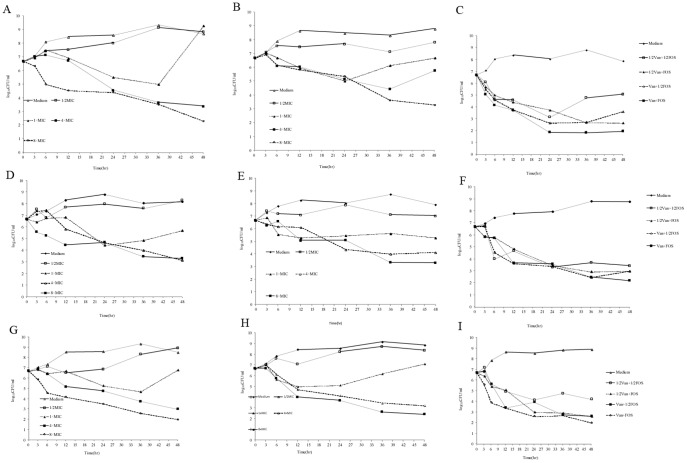
Time–kill kinetics of the methicillin-resistant *Staphylococcus aureus (MRSA)* strains KZ306 (A–C), ATCC43300 (D–F) and *Staphylococcus epidermidis* strain ATCC35984 (G–I). (A, D, G, Vancomycin alone, and B, E, H, fosfomycin alone; C, F, I, Combination with vancomycin (VAN) and fosfomycin (FOS). All experiments were performed in duplicate and the growth curve was plotted with average at each time point.

The MIC of VAN and FOS against KZ306, ATCC43300 and *S. epidermidis* strain ATCC35984 were 2 µg/mL and 128 µg/mL, respectively. For VAN and FOS alone, ½× MIC concentrations showed no bactericidal effects throughout the experiment (Fig. 1A and 1B). The inhibitory effect of 1× MIC of either drug alone persisted for 36 h; thereafter the colony numbers rebounded. Notably, strong bactericidal effects were seen at 4× and 8× MIC for both drugs (Fig. 1A and 1B).

To test the effects of the combination of VAN and FOS on KZ306, different combinations (½× MIC VAN +½× MIC FOS, 1× MIC VAN +½× MIC FOS, ½× MIC VAN +1× MIC FOS and 1× MIC VAN + 1× MIC FOS) were used in the time-kill assay. Six hours after administration, all combinations of the two drugs showed significant synergistic bactericidal effect, which lasted up to 24 h. Of note, the bactericidal effect of the combinations of ½× MIC VAN +½× MIC FOS and 1× MIC VAN +½× MIC FOS lasted up to 24 h and 36 h, respectively (Fig. 1C). However, the bactericidal effect of the combinations of ½× MIC VAN +1× MIC FOS and 1× MIC VAN +1× MIC FOS lasted up to 48 h (Fig. 1C). Similar results were obtained with MRSA strain ATCC43300 (Fig. 1D–1F) and *S. epidermidis* strain ATCC35984 (Fig. 1G–1I).

### 3.2 Changes in WBC and CRP after in vivo treatment of VAN or FOS or combination of both

**Figure 2:**
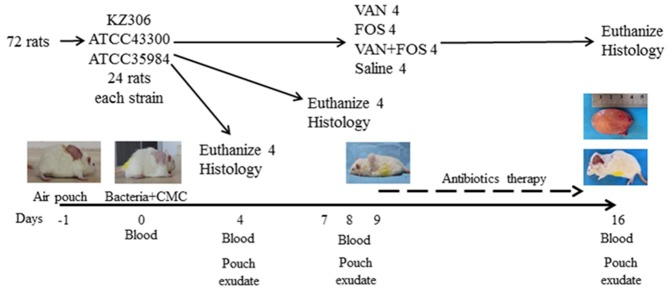
The experiment design employed in this study. The rat sodium carboxymethylcellulose (CMC)-pouch MRSA biofilm infection model was established in twenty-four rats for each strain. On day 0, the CMC pouch was formed by injecting CMC and MRSA strains KZ306, ATCC43300 or Staphylococcus epidermidis strain ATCC35984. On Days 4 and 8, four rats were euthanized, and the pouches were excised for histology examination. On Day 9 after the bacterial infection, the remaining 16 rats for each strain were randomly divided into four groups to receive antibiotics therapy or saline for 7 days. On Day 16, all rats were euthanized and pouches, blood samples and exudates from the pouches were collected for examination.

**Figure 3:**
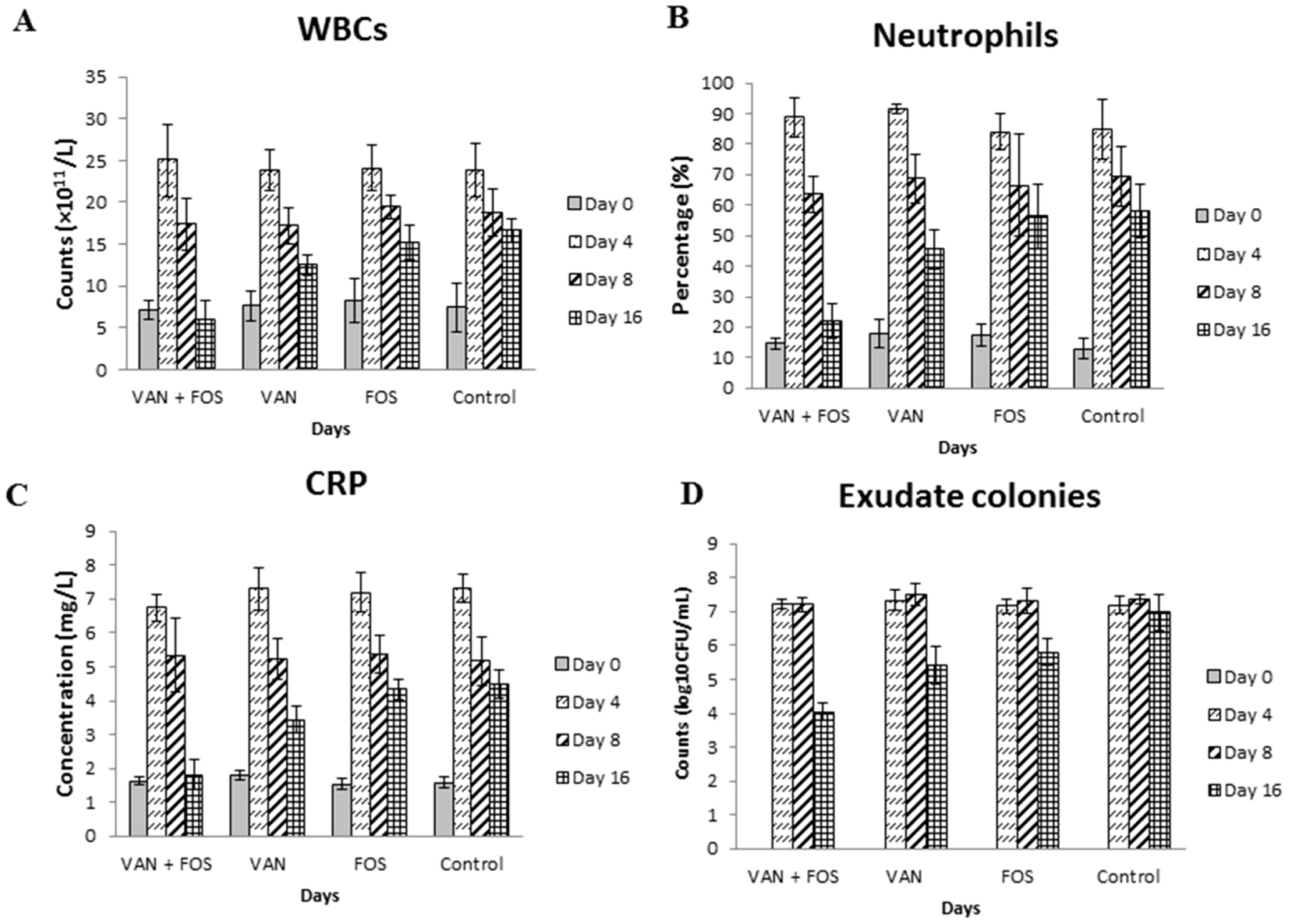
Changes in WBC counts (A), neutrophil percentage (B), CRP level (C) and colony counts in pouch exudates (D) after antibiotic therapy in animal biofilm model with KZ306 strain.

To evaluate the bactericidal effects of VAN and FOS *in vivo* on biofilm MRSA infection, we established a carboxymethyl cellulose (CMC)-pouch biofilm model in rats. The detailed protocol of the experiment is described in Fig. 2. The results of blood tests of animals injected with strains KZ306, ATCC43300, and *S. epidermidis* strain ATCC35984 and treated with antibiotics are illustrated in Fig. 3, S1 and S2 Figs., respectively. The results of blood tests in all thestrains were consistent. For example, in strain KZ306, the WBC counts, the percentages of neutrophils, and the CRP levels were significantly greater on Days 4 and 8 after biofilm formation, compared with the controls samples, suggesting that the infection model was successfully established (Fig. 3). On Day 8 after bacterial injection, all variables had decreased significantly (*P*<0.05) from the high levels seen on Day 4, but the levels were still higher than those before infection. However, there was no apparent difference among antibiotics groups. On Day 16, the WBC counts, neutrophil percentages and CRP levels in all groups were less than that on Day 8. More prominent reductions were observed in the combination group and all variables on Day 16 were close to the baselines. On day 16, while all variables in the VAN alone group showed no statistical difference from that in the control group, the levels of WBC counts and CRP values in the FOS alone group were significantly lower than that in control and VAN alone groups. More importantly, all variables in the combination groups of both strains were significantly reduced from that in the FOS alone and VAN alone groups.

The numbers of bacteria in pouch exudates from the MRSA-infected biofilm are also shown in Fig. 3, S1 and S2 Figs. For both strains examined, there were no significant differences in the colony counts between on Days 4 and 8 among all the groups. However, on Day 16, the colony counts were significantly decreased in all therapy groups compared to counts before therapy (*P*<0.05). The colony counts in the combination group were significantly lower than those in VAN and FOS alone groups (*P*<0.05). The blood test results and bacteria colony counts results suggested that combination therapy of VAN and FOS had a synergic effect on both MRSA strains and *S. epidermidis* strain, and was more effective in bactericidal activity than either antibiotic alone.

### 3.3 Histological changes after treatment of VAN or FOS or combination of both *in vivo*

**Figure 4:**
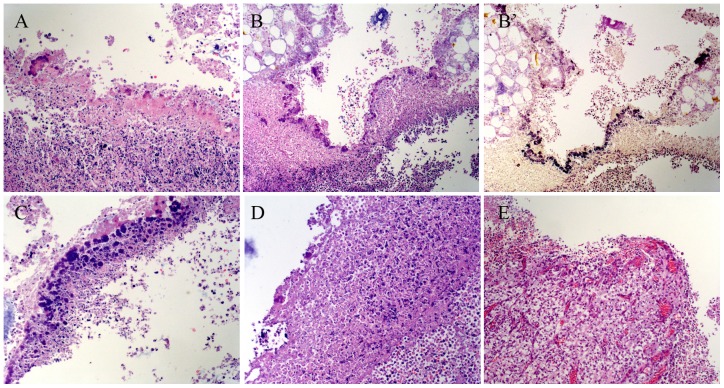
Histological changes in the pouch tissues. A, On Day 4 after infection (HE at 200× magnification), the pouch wall was made up of mostly necrotic tissue with scattered bacteria mass on its surface. B, On Day 8 (HE at 100× magnification), extensive necrotic tissue formed on the pouch surface with continuous bacteria colonies (black arrows), and inflammatory cell infiltration. B′, On Day 8 (Gram staining at 100× magnification), widespread Gram-positive bacterial colonies formed on the pouch surface at the same site showed in B (black arrows). C, On Day 16 (HE at 400× magnification), multilayer colonies formed on the pouch surface with more necrotic tissue in the control animals. A large number of inflammatory cells had infiltrated the necrotic tissues. D, On Day 16 (HE at 200× magnification), scattered colonies (black arrows) were observed in the necrotic tissues treated with either VAN or FOS. E, On Day 16 (HE at 200× magnification), bacterial colonies disappeared from animals receiving the combination therapy; newly formed granular tissues were observed.

Histological analysis of the pouch walls was completed using HE staining and Gram staining for both strains (Fig. 4). The bacteria were Gram-positive cocci at all the time points (Fig. 4B). Four days after bacterial injection, the pouch wall was made up of mostly necrotic tissue (Fig. 4A). There was only a small quantity of loosely scattered bacteria on the non-integrated surface. On Day 8, a large number of bacteria were lined up continuously on the surface of the necrotic tissue, indicating formation of a mature biofilm (Fig. 4B and 4B). On Day 16 (i.e. 8 days after antibiotic treatment), the pouches of the control group showed a further increase in bacterial mass and multilayer colonies attaching to the necrotic tissue surface were surrounded by large numbers of inflammatory cells (Fig. 4C). In rats treated with either FOS or VAN, there were a small number of scattered colonies existing on the surface of the pouch walls (Fig. 4D). On the contrary, in the rats treated with a combination of VAN and FOS, bacterial colonies were not found on the pouch walls, and a limited number of inflammatory cells infiltrated the biofilm (Fig. 4E). Notably, newly formed granulation tissues were observed; blood vessels growing into the wound and fibroblast cells implied that the wound was in a healing stage. These results indicated that the combination of VAN and FOS could eradicate MRSA, eliminate the biofilm, and control the infection.

## Discussion

In the present study, a rat CMC-pouch biofilm model was used to evaluate the *in vivo* bactericidal effects of VAN and FOS administered alone or in combination against biofilm-infected MRSA and *S. epidermidis*. Synergistically bactericidal effects were observed in the combination therapy. For both MRSA strains and *S. epidermidis* strain, in comparison with the control groups and either VAN or FOS alone, the combination groups had lower colony counts in the pouch exudates and lower WBC counts, neutrophils and CRP levels in peripheral blood. Furthermore, the histological analysis of the biofilm tissues from the combination group indicated a loss of biofilm structure, a marked decrease in necrosis, and formation of granular tissue, suggesting the beginning of the healing process. These results indicated that the combination of VAN and FOS could eliminate MRSA and *S. epidermidis* more efficiently in biofilms, controlling microbe-induced inflammation.

It has been reported that the combination of VAN and FOS could synergistically eliminate MRSA embedded in *in vitro* biofilms [2], [19]. The present study was designed to test whether the synergic bactericidal effect *in vitro* could be demonstrated *in vivo*. The rat subcutaneous CMC-pouch biofilm model used in this study was initially used to evaluate the efficacy of antibiotics against cystic granuloma and to explore the effect of antibiotics on biofilm-infected bacteria [11], [22]. The reliability of the model has been proven, and one of its advantages is permitting frequent sampling for the examination of the anti-bacterial activity and inflammatory process in the pouch tissues [11], [22]. The model has been used not only to test the effect of antibiotics permeating into microbes in biofilms, but also to elucidate the structural formation and the inflammatory response of the biofilm [2], [22]. A notable modification in this study was that we changed the time for beginning of antibiotics therapy from Day 5 to Day 9 after infection. The resistance of biofilm-microbes to antibiotics varies with the growth status of the biofilm. Microbes living in mature biofilms have greater tolerance for antibiotics than those in newly formed biofilms; therapy becomes more difficult with greater quantities of mature biofilms [23]. In order to confirm the synergic activity of the two antibiotics in the long-term, chronic and more mature biofilm, administration of antibiotics began on Day 9 after the infection. We assumed that the biofilm on Day 9 was more mature, and the animal models were more likely to mimic human chronic infections. Thus, the results would be more practical for clinical use. In fact, histological evidence indicated that the MRSA biofilms were more integrated and mature on Day 8 than that on Day 4. On day 8, the biofilm-like bacterial mass on the surface of necrotic tissues was more extensive, continuous, and integrated. In addition, the levels of WBC, neutrophils and CRP had decreased by Day 8, indicating that the rat CMC-pouch biofilm infection model was well established and close to mimicking clinical infectious biofilm conditions.

Combination therapy with two or more antibiotics is believed to be more effective to eliminate biofilm-microbes because of different tolerance mechanisms of microbes embedded in the different locations of biofilm. Since various antibiotics possess different anti-bacterial mechanisms and various bacteria possess different resistant mechanisms, it is almost impossible to use a single antibiotic with a specific bactericidal mechanism to completely eradicate all of the biofilm-bacteria. Combination therapy with two or more antibiotics with different bactericidal mechanisms can synergistically and complementarily eradicate biofilm-bacteria [24]. The theory of the mutant selection window (MSW) provides an explanation for the advantage of the combination therapy with multiple antibiotics: MSW can be closed to prevent mutant selection by combining multiple antibiotics that have different bactericidal mechanisms, thus increasing the likelihood of killing bacteria and, simultaneously, reducing the disadvantageous side-effects of the antibiotics [25]. In the present study, VAN or FOS alone at ½× MIC had no bactericidal effect. Bactericidal effects were observed for the single drugs at 1× MIC at 24 h, but regrowth occurred after another 24 h. A long-term and complete inhibitory effect was only shown for higher concentrations (4×, 8× MIC VAN and 8× MIC FOS) of the single antibiotics. VAN or FOS alone at low MIC could only provide limited anti-bacterial effect. However, the combination therapy provided long-term and complete anti-bacterial efficacy at low concentrations (½× MIC VAN +1× MIC FOS and 1× MIC VAN +1× MIC FOS). Therefore, it is likely that the combination therapy could provide a better therapeutic outcome, further highlighting the importance of using the combination therapy to obtain maximal effects.

Not all the antibiotics would exert antibacterial effects synergistically; some might even function antagonistically. The phenomenon was observed in the combination therapy of rifampin and VAN [26]. Rifampin is a potential antibiotic against biofilm infection since it has a strong ability to penetrate biofilms and inhibit surface adherence of microbes, but can easily induce tolerance when used alone. Therefore, rifampin is usually used in combination with other antibiotics [25], [27], [28]. However, controversy has arisen with regard to the efficacy of VAN combined with Rifampin in therapy against biofilm-infections [10], [15], [25], [26], [28]. FOS alone has a bactericidal effect both *in vivo* and *in vitro*, but MRSA easily develops tolerance, making use of FOS unfeasible for clinical settings [14]. FOS exhibits favorable synergistic effects on MRSA biofilm when used in combination with other antibiotics [2], [14], [19], [22]. Furthermore, combination therapy using FOS and VAN reduces the toxic effect of VAN on kidneys, while providing a great benefit for patients with renal insufficiency [29]. FOS plays a major role in the synergistic effect of combination therapy using VAN and FOS both *in vitro* and *in vivo*. FOS interferes with the biosynthesis of bacterial peptidoglycan and thereby alters the permeability of cell membranes by inhibiting the synthesis of cell walls at an early stage and providing a pathway for other antibiotics to act on the bacteria [22]. In addition, FOS itself is a low molecular agent, and can penetrate biological membranes, including mature biofilms [13]. Furthermore, the bactericidal activity of FOS is stronger in anoxic environments (i.e. biofilms) [30]. As a result, FOS has good efficacy for eliminating bacteria both on the surface and within biofilms [14]. A combination of FOS and VAN has a synergistic bactericidal effect on S. aureus [17], [18] as well as MRSA strains embedded in in vitro biofilm models [2], [19]. Our results from the present study verified the synergistic effect on MRSA in an in vivo mature biofilm model. In our animal study, the WBC counts, neutrophils, CRP in peripheral blood samples and exudate colonies reached maximum on Day 4 after infection and decreased significantly on Day 9 after antibiotics therapy in treatment groups. With the H&E staining, while bacteria mass and necrotic tissue could be observed on the pouch surface on Day 4, the extensive necrotic tissue with continuous bacteria colonies was shown on Day 8. On Day 9 post therapy, the bacterial colonies were eliminated by the antibiotics therapy, especially in the combination group. When compared to control and single antibiotic groups, the synergistic effects were observed in colony counts in the pouch exudates, WBC counts, neutrophils, and CRP in peripheral blood. Furthermore, the synergistic effects were also demonstrated by morphological changes. HE staining and Gram staining at the same tissue sites clearly demonstrated that the bacterial colonies were eliminated by the combination therapy. Thus, the combination therapy of VAN and FOS may be clinically effective for treating patients with biofilm-associated MRSA infections.

In summary, in the present *in vivo* study with a rat CMC-pouch biofilm MRSA model, we demonstrated the synergic bactericidal efficacy of VAN in combination with FOS. Our findings will provide a basis for clinicians to choose an effective treatment of chronic MRSA infection. The validity of the findings should be confirmed by prospective clinical studies on the therapy of chronic MRSA infections.

## Supporting Information

S1 Fig **Changes in WBC counts (A), neutrophil percentage (B), CRP level (C) and colony counts in pouch exudates (D) after antibiotic therapy in animal biofilm model with MRSA strain ATCC43300.**
(TIF)

S2 Fig **Changes in WBC counts (A), neutrophil percentage (B), CRP level (C) and colony counts in pouch exudates (D) after antibiotic therapy in animal biofilm model with** ***Staphylococcus epidermidis*** **strain.**
(TIF)
