# Supplementary figures and images for: Efficacy of Combined Vancomycin and Fosfomycin against Methicillin-Resistant Staphylococcus aureus in Biofilms In Vivo
Source: PLoS One. 2014 Dec 31;9(12):e113133. doi: 10.1371/journal.pone.0113133 (PMC4281042; doi:10.1371/journal.pone.0113133)

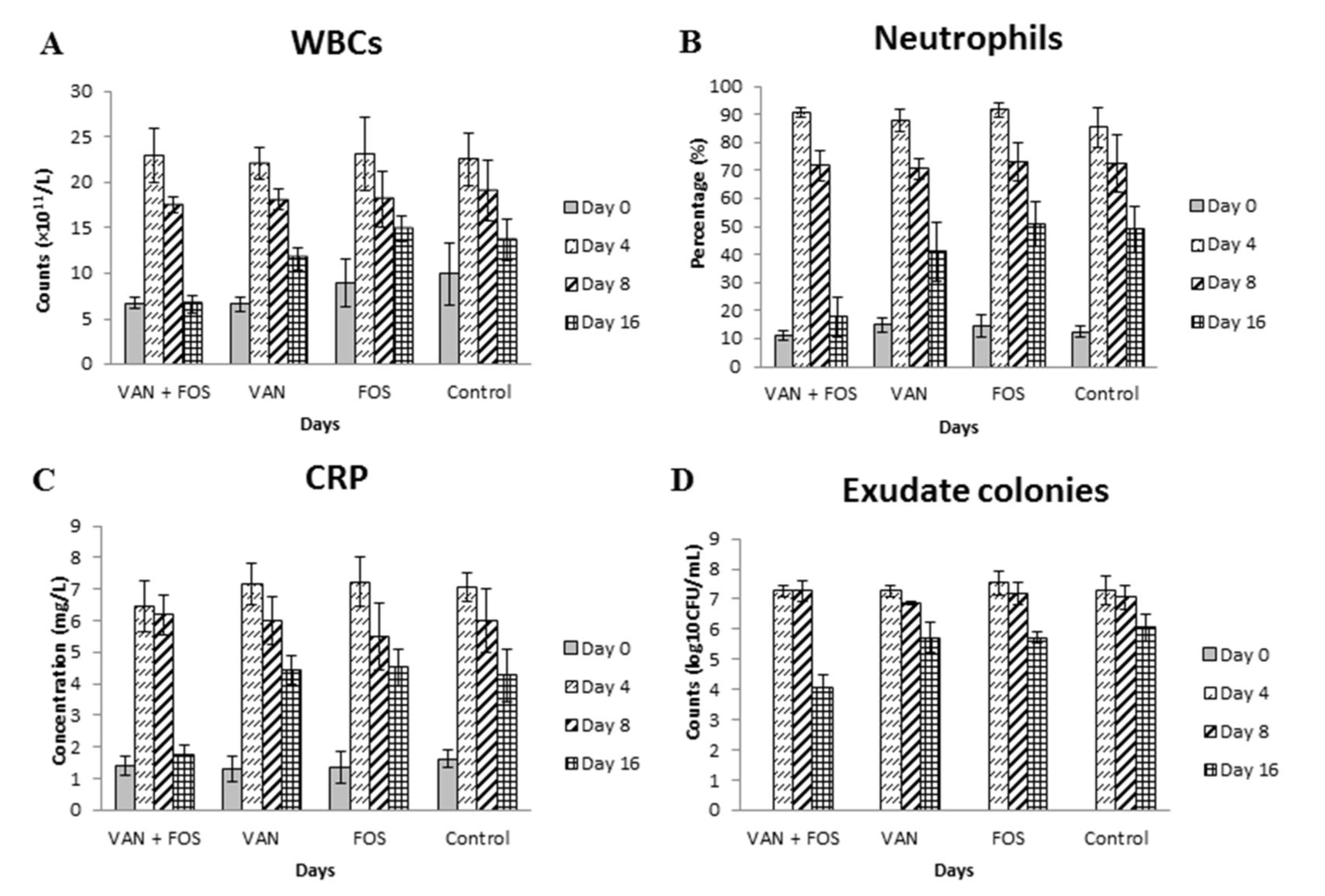

Supplement: S1 Fig — Changes in WBC counts (A), neutrophil percentage (B), CRP level (C) and colony counts in pouch exudates (D) after antibiotic therapy in animal biofilm model with MRSA strain ATCC43300. (TIF) [file pone.0113133.s001.tif]

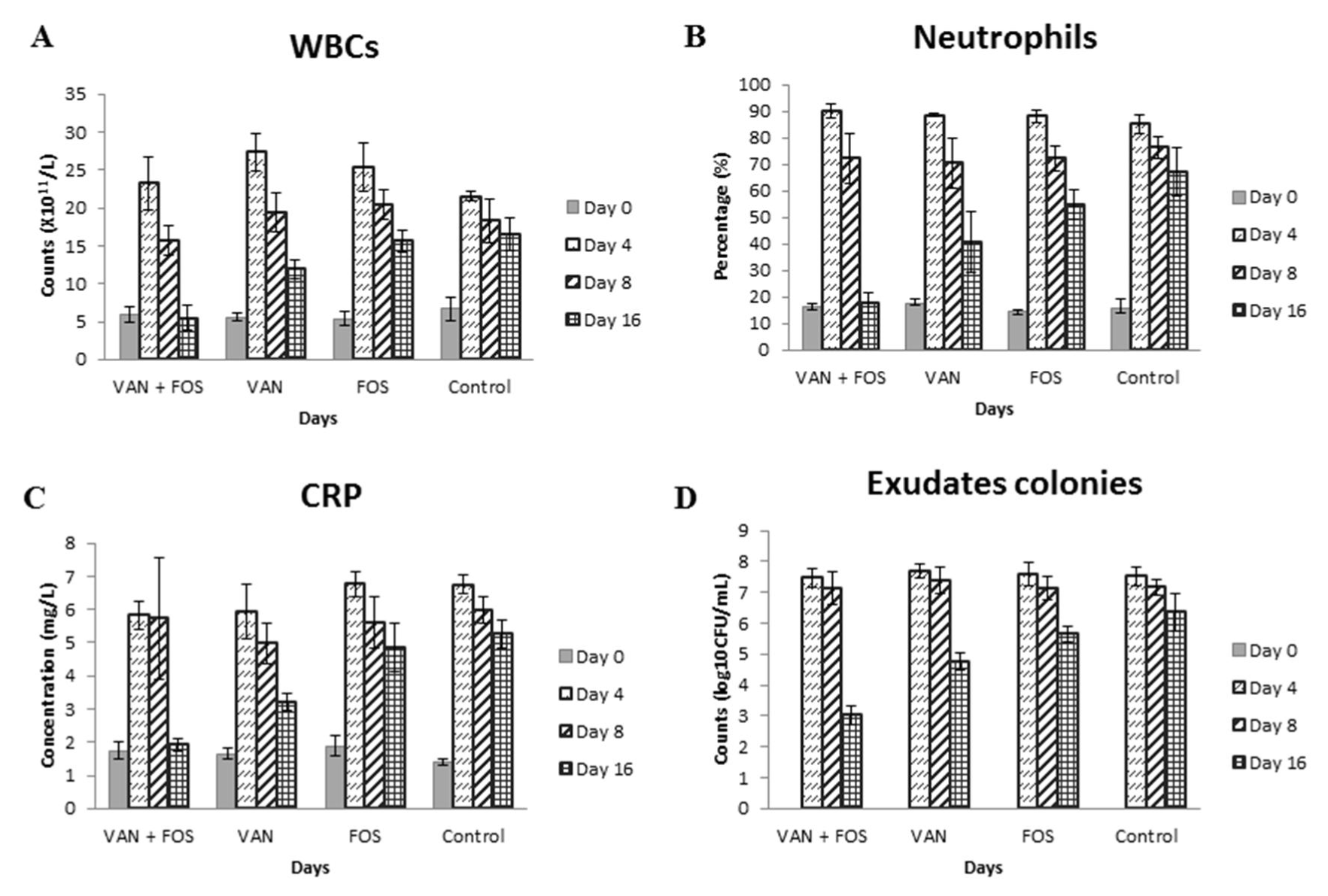

Supplement: S2 Fig — Changes in WBC counts (A), neutrophil percentage (B), CRP level (C) and colony counts in pouch exudates (D) after antibiotic therapy in animal biofilm model with Staphylococcus epidermidis strain. (TIF) [file pone.0113133.s002.tif]
